# Early first trimester peripheral blood cell microRNA predicts risk of preterm delivery in pregnant women: Proof of concept

**DOI:** 10.1371/journal.pone.0180124

**Published:** 2017-07-10

**Authors:** Edward E. Winger, Jane L. Reed, Xuhuai Ji

**Affiliations:** 1 Laboratory for Reproductive Medicine and Immunology, San Francisco, CA, United States of America; 2 Stanford University, Human Immune Monitoring Center, Stanford, CA, United States of America; Hopital Robert Debre, FRANCE

## Abstract

**Objective:**

We investigated the capacity of first trimester peripheral blood mononuclear cell (PBMC) microRNA to determine risk of spontaneous preterm birth among pregnant women.

**Study design:**

The study included 39 pregnant women with the following delivery outcomes: 25 with a full term delivery (38–42 weeks gestation) 14 with spontaneous preterm birth (<38 weeks gestation). Of the 14 women experiencing spontaneous preterm birth, 7 delivered at 34-<38 weeks gestation (late preterm) and 7 delivered at <34 weeks gestation (early preterm). Samples were collected at a mean of 7.9±3.0 weeks gestation. Quantitative rtPCR was performed on 30 selected microRNAs. MicroRNA Risk Scores were calculated and Area-Under the Curve-Receiver-Operational-Characteristic (AUC-ROC) curves derived.

**Results:**

The AUC-ROC for the group delivering preterm (<38 weeks) was 0.95 (p>0.0001). The AUC-ROC for early preterm group (<34 weeks) was 0.98 (p<0.0001) and the AUC-ROC for the late preterm group (34-<38 weeks) was 0.92 (p<0.0001).

**Conclusion:**

Quantification of first trimester peripheral blood PBMC MicroRNA may provide sensitive and specific prediction of spontaneous preterm birth in pregnant women. Larger studies are needed for confirmation.

## Introduction

Spontaneous preterm birth (SPTB) is the most common cause of neonatal morbidity and mortality [1]. More than one in ten babies worldwide are born with SPTB with many facing lifelong disability [2] Prematurity is the second leading cause of death in children under five years of age and the single most important cause of death in the first month of life [3]. Better described as a syndrome, SPTB is the clinical manifestation of multiple and widely divergent antecedent conditions. While preeclampsia is thought to be a disorder arising in the placental bed, Romero et al. suggest only a subset of preterm births appear to share this origin [4, 5]. Identification of common features identifiable early in pregnancy would thus seem quite challenging.

The annual cost of SPTB in the United States alone is at least 26.2 billion dollars [6]. While most studies have focused on early preterm births (<34 weeks gestation), their relative incidence constitutes only about one fourth of all preterm births. The American College of Obstetrics and Gynecology now recognizes increased risk during the early part of the conventional period designated as term delivery [7]. Beta et al. found that pregnancies delivering at week 37 have twice the risk of complications than those delivered at week 38 [8]. We, therefore, chose to extend our definition of late preterm birth to <38 weeks gestation as this time point is a more clinically relevant for identification of at-risk pregnancies.

Some methods for calculating the risk of SPTB are based on clinical criteria alone [9]. Various investigators have included maternal age, height, weight, racial origin, smoking status, spontaneous or assisted conception and obstetric history among predictive factors. The addition of cervical echography has enhanced the predictive power of these clinical criteria [10]. Biochemical tests of maternal serum constituents have also been added to predictive models. While levels of PAPP-A have been found diminished in maternal plasma during the first trimester [11], a variety of other biomarkers studied including free β-hCG have not been found predictive [12]. Development of a single diagnostic assay, particularly one implemented at an early time point, remains an important goal [13, 14].

MicroRNAs are ubiquitous small non-coding RNA molecules of about 22 bases. They regulate translation of mRNAs into protein. MicroRNAs have attracted great interest for their potential as clinical tools in diagnosis and monitoring. Their release from diseased tissues into plasma permits non-invasive monitoring by simple venipuncture, the “liquid biopsy.” Extraction and quantification of microRNA from plasma has been used to study a wide variety of disorders such as cancer, autoimmune, inflammatory, cardiovascular and neurologic diseases [15]. However, plasma microRNA collected during the first trimester has been examined as a potential marker for preeclampsia without success [16]. We know of no corresponding studies in SPTB.

Instead of peripheral blood plasma, we chose to examine peripheral blood mononuclear cell (PBMC) microRNA. In our previous studies, we found that first trimester PBMC microRNA provides sensitive and specific prediction of preeclampsia at 6–8 weeks gestation [17, 18]. In these studies, quantification of a panel of 30 microRNAs as established in our previous studies was performed. In our present study, we wished to widen our first trimester microRNA investigations from preeclampsia to SPTB prediction.

## Materials and methods

### Study population

The patients were seen at a high risk medical clinic for recurrent pregnancy loss and infertility located in San Jose, California, USA. This patient population had an elevated incidence of adverse pregnancy outcome. Patients had a 17% preterm delivery rate and a 6% preeclampsia rate among singleton deliveries. Sixty-nine percent of the patients tested positive for one or more of the following autoimmune markers: antiphospholipid antibodies, antinuclear antibodies and/or anti DNA antibodies. Thirty-five percent of the patients entered the clinic with a prior history of recurrent (≥3) miscarriage and 21% of the patients entered the clinic with a prior history of two or more IVF failures (often diagnosed as “unexplained infertility”) at other centers. Many of the known preterm risk factors such as cervical abnormalities, infection, maternal disease, etc. had been addressed and treated prior to patient registration at the clinic.

### Patient selection and study design

#### Sample collection

This was a single-center, retrospective study. The investigation involved a retrospective analysis of frozen, Trizol-stabilized blood samples stored from September 2012 to September 2015. One hundred ninety-two deliveries were available for study within the allotted time frame. All pregnancies had been completed by the time of sample analysis. As all patients had been seen at a single clinic, variables inherent between patient groups and specimen collection, handling and cell preparation, were eliminated. Both full term and preterm samples were collected and prepared by the same clinical and laboratory personnel, stored in the same ultra-low freezer, under identical conditions. All were collected in sodium heparin tubes (BD Vacutainer tubes, Becton-Dickinson, Franklin Lakes, New Jersey, USA) and stored overnight at room temperature before processing at the Laboratory for Reproductive Medicine and Immunology (San Jose, USA). PBMCs were prepared by Ficoll-hypaque destiny gradient centrifugation as performed in our previous studies [17, 18]. The PBMCs were stored at -80^°^C after addition of Trizol according to the manufacturer’s instructions (Thermo-Fisher, USA).

#### Inclusion criteria

For study inclusion, all samples were from patients that met the following criteria: 1) index cycle between September 2012 and September 2015; 2) frozen Trizol-treated blood sample available from the first trimester; 3) singleton delivery; 4) spontaneous delivery, if delivery was preterm; 5) delivered baby with no obvious birth defects. Deliveries were excluded if they were post term (>42 weeks) and in cases where the clinic failed to receive delivery details. Using these combined criteria, 54 candidate deliveries were eligible for study inclusion (34 full term and 20 preterm deliveries). These were then submitted blinded to the laboratory for inclusion on a 48 sample PCR chip. All twenty of the preterm delivery samples and 28 randomly selected out of the 34 term delivery samples were designated for PCR quantification. Ten samples initially designated for inclusion on the PCR chip failed to meet quality requirements as previously described [17,18] and were replaced by the remaining 6 samples for a total of 44. Five samples failed to signal adequately on PCR amplification. This brought the total number of useable sample results to 39 (25 full term and 14 preterm).

#### Patient consent

The study was a retrospective analysis using clinical data from patient charts and specimens frozen and stored as PBMC pellets. Venous blood was obtained from women who had given written informed consent to provide samples for research. Institutional Review Board approval for retrospective use of the specimen archive and consent form had been obtained (Western Institutional Review Board (WIRB), Study Number 1151255, Pro. Number 20142368) [17, 18]. Patient identifying information was maintained in accordance with HIPAA requirements.

#### Specimen transport

Specimens prepared as PBMC pellets were stored in Trizol at -80°C and transported on dry ice from the Laboratory for Reproductive Medicine and Immunology (San Jose, California, USA) to the Stanford Human Immune Monitoring Center (Stanford, California, USA) identified only by date and accession number. The Center was blinded to clinical outcome prior to the completion of testing. Samples were analyzed by quantitative reverse transcription polymerase chain reaction (quantitative rtPCR) as previously performed [17, 18].

#### Equivalency of patient groups

As shown in Table 1, the full term and preterm delivery groups in our analysis were generally similar in terms of history, age, test results and prior pregnancy history. In addition, all women included in the study were known to be of a mature reproductive age (> 28 years), were in stable partner relationships and were actively seeking pregnancy. There were with no active smokers, drinkers, recreational drug users or teenage or single mothers included in the patient population. Lastly, all patients were being seen at a high risk, self-pay, specialist clinic and so they likely represented a middle to upper class socioeconomic group. Except for factors directly associated with preterm delivery itself (birthweight and PROM), the only patient parameters found to be statistically different between the outcome groups, were high Body Mass Index (p = 0.01) and non-white race (p = 0.01).

**Table 1:** Study population.

	Full term delivery (38–42 weeks)	SPTB (<38 weeks)	SPTB subsets:		P value (Full term to SPTB)
Patient history:			Late SPTB: (34-<38 weeks) **	Early SPTB(<34 weeks)	
**Number of patients:**	25	14	7	7	
**Age (yrs)**	36.5±4.7	39.3±6.4	39.3±5.2	39.3±7.8	0.13
**Race:**					
**White (%)**	84% (21/25)	64% (9/14)	57% (4/7)	71% (5/7)	0.01
**Asian (%)**	8% (2/25)	7% (1/14)	14% (1/7)	0% (0/7)	1
**Middle East (%)**	4% (1/25)	7% (1/14)	14% (1/7)	0% (0/7)	1
**Hispanic**	4% (1/25)	0%(0/14)	0% (0/7)	0% (0/7)	1
**Black (%)**	0% (0/25)	7% (1/14)	0% (0/7)	14% (1/7)	0.36
**Other/mixed (%)**	0% (0/25)	14% (2/14)	14% (1/7)	14% (1/7)	0.13
**Prior livebirth**	24% (6/25)	29% (4/14)	43% (3/7)	14% (1/7)	1
**Prior preterm delivery**	0% (0/25)	14% (2/14)	14% (1/7)	14% (1/7)	0.12
**Prior miscarriage**	72% (18/25)	50% (7/14)	57% (4/7)	43% (3/7)	0.3
**BMI (Mean ± SD)**	22.4±3.9	26.3±5.4	27.5±4.3	25.1±6.4	0.01
**Antiphospholipid antibody +**	16% (4/25)	43% (6/14)	29% (2/7)	57% (4/7)	0.12
**Antinuclear antibody +**	4% (1/25)	14% (2/14)	0% (0/7)	29% (2/7)	0.29
**Anti-DNA antibody +**	8% (2/25)	0% (0/14)	0% (0/7)	0% (0/7)	0.53
**ART conception (%)**	40% (10/25)	57% (8/14)	43% (3/7)	71% (5/7)	0.33
**Donor egg cycle (%)**	8% (2/25)	29% (4/14)	29% (2/7)	29% (2/7)	0.16
**Chlamydia history (%)**	12% (3/25)	7% (1/14)	0% (0/7)	14% (1/7)	1
**Corticosteroid therapy (%)**	48% (12/25)	50% (7/14)	57% (4/7)	43% (3/7)	1
**Anticoagulant therapy (%)**	88% (22/25)	86% (12/14)	86% (6/7)	86% (6/7)	1
**Gest. age at sampling (weeks)**	8.3±2.8	7.2±3.1	7.6±3.2	6.8±3.2	0.26
**Gest. age delivery (weeks)**	39.2±1.2	33.9±2.9	36.3±1.1	31.5±1.9	<0.0001
**Birthweight (g)**	3278±538	2304±613	2701±516	1908±423	<0.0001
**Preeclampsia (%)**	12% (3/25)	29% (4/14)	14% (1/7)	43% (3/7)	0.23
**PROM (%)**	4% (1/25)	57% (8/14)	57% (4/7)	57% (4/7)	0.0004
**IUGR (%)**	4% (1/25)	0% (0/14)	0% (0/7)	0% (0/7)	1

**Three of our 7 “late preterm” deliveries were delivered at 37 to 38 weeks gestational age

#### MicroRNA selection

Each of the samples was analyzed using 30 microRNAs by rtPCR selected for clinical utility in and performed according to the protocol in our previous studies [17, 18]. These microRNAs included: hsa-miR-340-5p, -424-5p, -33a-5p, -7-5p, -1229, -1267, -671-3p, -1, -133b, -144-3p, -582-5p, -30e-3p, -199a-5p, -199b-5p, -210, -221-5p, -575, -301a-3p, -148a-3p, -193a-3p, -219-5p, -132, -513a-5p, -1244, -16, -146a, —155, -181a, -196a and -223. Quantification was recorded as the PCR Ct. (Ct or Cycle threshold, values correspond inversely to the microRNA concentration where Ct represents the number of PCR amplification cycles required to reach a detection threshold).

#### Assignment of samples to training and validation sets

**Fig 1:**
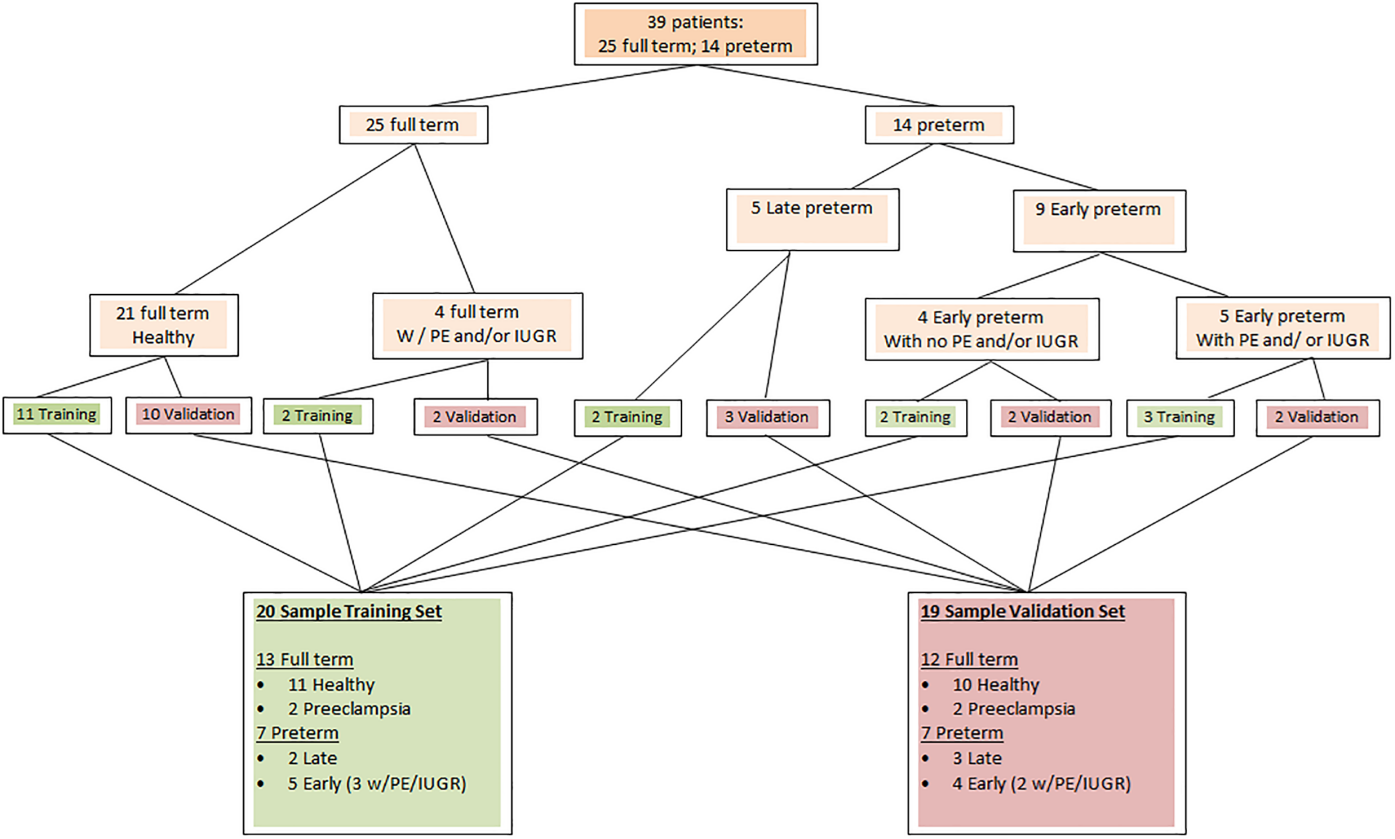
Assignment of samples to training and validation sets. The 39 samples that met the entry criteria for the study were randomly assigned to “training” and “validation” sets using group randomization software (Medcalc® version16.1.2). To assure 25 full term and 14 preterm deliveries were sorted into subgroups. The 25 full term deliveries were divided into two subgroups: 1) uncomplicated full term deliveries 2) full term deliveries with coexisting IUGR and/or preeclampsia. The 14 preterm patients were divided into three subgroups 1) Uncomplicated early preterm deliveries 2) early preterm with co-existing IUGR and/or preeclampsia 3) Uncomplicated late preterm deliveries (there were no late preterm deliveries with co-existing IUGR or preeclampsia. Subgroup randomization helped to overcome bias inherent in dividing very small population. Once created, the training set population (20 samples) was used to develop the microRNA scoring system. This MicroRNA Scoring System was later verified using the 19 sample validation set.

The 39 samples successfully quantified were randomly assigned to “training” and “validation” sets using the group randomization software from the Medcalc suite version 16.1.2 (Medcalc® version16.1.2, Ostend, Belgium). To provide more even distribution of delivery outcome between Training and Validation sets given small sample size, 25 full term and 14 preterm deliveries were randomized from smaller subgroups defined by the presence of preeclampsia and/or IUGR (Fig 1).

#### Development of the microRNA scoring system using the training set

MicroRNA Ct values were analyzed by Medcalc® software generating an AUC-ROC (Area Under the Curve-Receiver Operating Characteristic), p value and Youden Index J Associated Criterion Value for each of microRNAs in the training set. MicroRNAs where the p value was less than 0.05 were included in the panel. Eight microRNAs met this criterion comprising miR-148a, -301a, -671, -181a, -210, -1267, -223, and -340 (Table 2). Ct values less than the Youden Value (“cut-off” value) for each of the microRNAs in the panel were given a score of “1”. The panel and respective cutoff values determined from analysis of the training set were then applied to the validation set and its AUC-ROC calculated. The individual microRNA scores for each sample were summed to create the Risk Score.

**Table 2:** Development of the microRNA scoring system using the training set.

#	Selected for panel	MicroRNA	AUC-ROC	p value	Youden J Associated Criterion Value	95% Confidence interval	Sensitivity	Specificity	Sample size	Positive group	Negative group
**1**	**x**	**148a-3p**	1	0.0001	≤28.04	≤27.93 to ≤28.04	100	100	11	6 (54.55%)	5 (45.45%)
**2**	**x**	**301a-3p**	0.9	0.0001	≤25.08	≤24.64to ≤25.08	100	69.23	20	7 (35.00%)	13 (65.00%)
**3**	**x**	**671-3p**	0.98	0.0001	≤28.80	≤28.03 to ≤28.75	100	88.89	14	5 (35.71%)	9 (64.29%)
**4**	**x**	**181a**	0.84	0.0005	≤25.84	≤24.61 to ≤26.91	85.71	72.73	18	7 (38.89%)	11 (61.11%)
**5**	**x**	**210**	0.81	0.002	≤21.04	≤19.70 to ≤22.19	71.43	84.62	20	7 (35.00%)	13 (65.00%)
**6**	**x**	**1267**	0.79	0.006	≤14.31	≤14.15to ≤15.47	85.71	76.92	20	7 (35.00%)	13 (65.00%)
**7**	**x**	**223**	0.77	0.02	≤12.33	≤9.95 to ≤12.61	85.71	61.54	20	7 (35.00%)	13 (65.00%)
**8**	**x**	**340-5p**	0.79	0.02	≤21.31	≤20.55to ≤23.01	57.14	100	20	7 (35.00%)	13 (65.00%)
**9**	** **	**16**	0.76	0.06	≤13.49	≤13.12 to ≤15.31	85.71	76.92	20	7 (35.00%)	13 (65.00%)
**10**	** **	**193a-3p**	0.75	0.08	≤26.67	≤25.00 to ≤29.78	71.43	75	15	7 (46.67%)	8 (53.33%)
**11**	** **	**132**	0.73	0.1	≤24.74	≤23.25 to ≤29.33	71.43	76.92	20	7 (35.00%)	13 (65.00%)
**12**	** **	**133b**	0.73	0.1	≤23.88	≤22.85to ≤27.48	71.43	76.92	20	7 (35.00%)	13 (65.00%)
**13**	** **	**30e-3p**	0.67	0.19	≤16.80	≤16.51 to ≤16.80	100	46.15	20	7 (35.00%)	13 (65.00%)
**14**	** **	**146a**	0.68	0.2	≤17.20	≤16.37 to ≤18.82	71.43	69.23	20	7 (35.00%)	13 (65.00%)
**15**	** **	**1244**	0.66	0.28	≤23.56	≤22.84 to ≤26.64	71.43	75	19	7 (36.84%)	12 (63.16%)
**16**	** **	**7-5p**	0.64	0.36	>28.47	>25.4 to >30.79	71.43	62.5	15	7 (46.67%)	8 (53.33%)
**17**	** **	**582-5p**	0.7	0.4	≤29.13	≤27.77 to ≤29.70	80	75	9	5 (55.56%)	4 (44.44%)
**18**	** **	**575**	0.67	0.43	≤27.34	≤23.94 to ≤27.34	100	66.67	12	6 (50.00%)	6 (50.00%)
**19**	** **	**199b-5p**	0.67	0.53	>26.50	>26.06 to >29.70	83.33	66.67	9	6 (66.67%)	3 (33.33%)
**20**	** **	**1229**	0.67	0.62	≤26.75	≤25.50 to ≤26.75	100	66.67	7	4 (57.14%)	3 (42.86%)
**21**	** **	**155**	0.51	0.97	≤14.13	≤12.11 to ≤14.62	28.57	46.15	20	7 (35.00%)	13 (65.00%)
**22**	** **	**196a**	0.5	1	>23.79	>22.49 to >26.72	100	50	9	5 (55.56%)	4 (44.44%)
**23**	** **	**1**	NA	NA	NA	NA	NA	NA	NA	NA	NA
**24**	** **	**144-3p**	NA	NA	NA	NA	NA	NA	NA	NA	NA
**25**	** **	**199a-5p**	NA	NA	NA	NA	NA	NA	NA	NA	NA
**26**	** **	**219-5p**	NA	NA	NA	NA	NA	NA	NA	NA	NA
**27**	** **	**221-5p**	NA	NA	NA	NA	NA	NA	NA	NA	NA
**28**	** **	**33a-5p**	NA	NA	NA	NA	NA	NA	NA	NA	NA
**29**	** **	**424-5p**	NA	NA	NA	NA	NA	NA	NA	NA	NA
**30**	** **	**513-5p**	NA	NA	NA	NA	NA	NA	NA	NA	NA

Using the 20 sample training set, an AUC-ROC was calculated for each of the 30 microRNAs used in the study.

MicroRNA where the p value was less than 0.05 were included in the panel.

“NA” represents microRNA for which an insufficient number of samples signaled to generate a ROC curve.

Eight microRNAs were included in the scoring panel: microRNA 148a, -301a, -671, -181a, -210, -1267, -223, and microRNA -340.

A cutoff value for each of these microRNAs was concurrently determined as the Youden Index J Associated Criterion Value.

Cutoff values for all eight microRNAs were then used to verify the eight-microRNA risk score for individual patients in the validation set.

## Results

### Verification of the microRNA Risk Score algorithm using the validation set

Using combined score for the 19 patient samples (12 full term; 7 preterm) an AUC-ROC of 0.95 was calculated for the validation set (p<0.0001). The predictive value of the microRNA Risk Score for preterm birth was, therefore, deemed confirmed.

### Application of Risk Score to the 39 patient population

**Fig 2:**
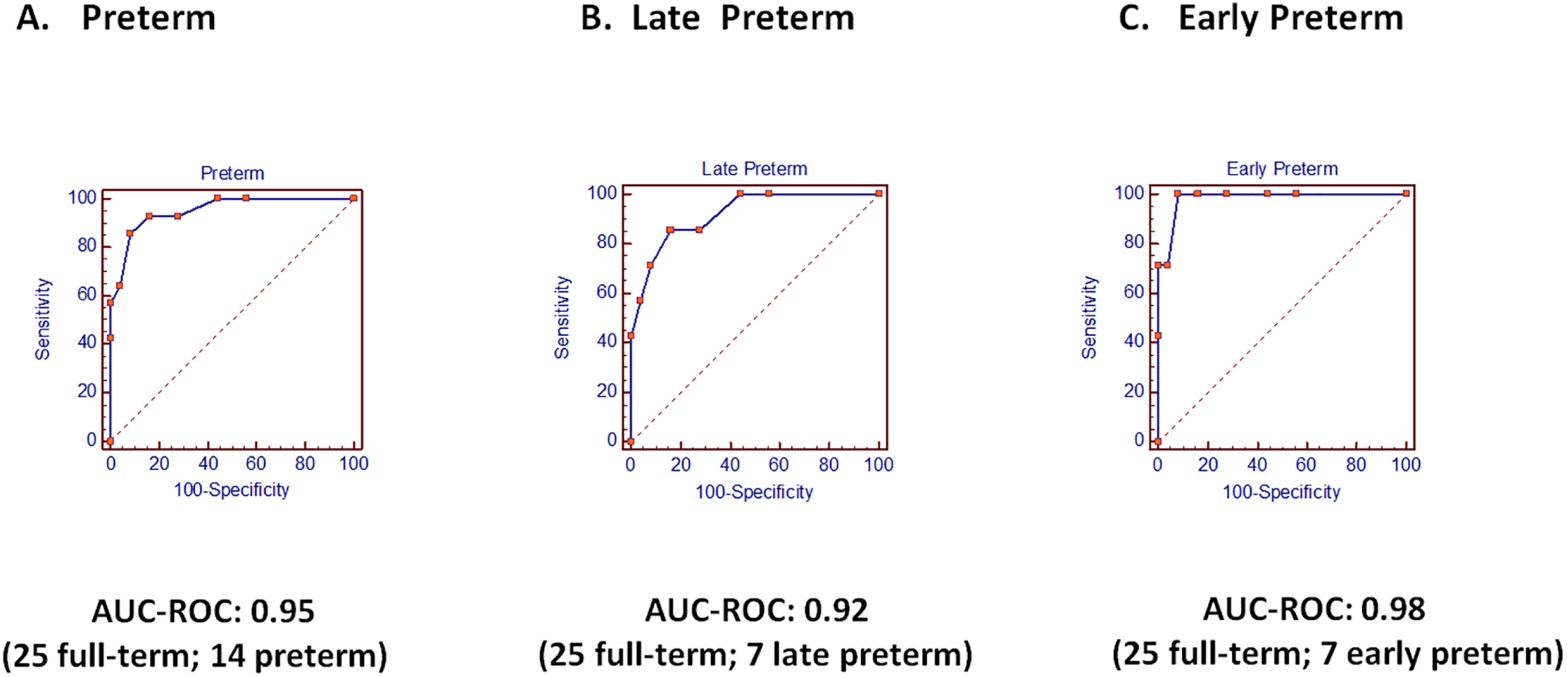
AUC-ROC curves for microRNA Risk Score prediction of spontaneous preterm birth.

After confirming the predictive value of Risk Score algorithm using the validation set, we then applied the 8 microRNA scoring system to the larger 39 patient study population (Table 3). AUC-ROC values were calculated for the different subgroups using the respective Risk Scores. The AUC-ROC value for all SPTB deliveries (<38 weeks) was 0.95 (p>0.0001). The AUC-ROC value for the early SPTB deliveries (<34 weeks) was 0.98 (p<0.0001) and the AUC-ROC value for the late SPTB deliveries (34-<38 weeks) was 0.92 (p<0.0001); (Table 4; Fig 2). When patient samples were divided by gestational age range at the time of specimen collection (5–6 weeks, 7–9 weeks, and 9–13 weeks), AUC-ROC values for each time range was found to be 1.00, 0.95 and 0.88, respectively. Due to clinical interest in in the time range around 10 weeks gestation (when early pregnancy studies such as NIPT or Non-invasive Prenatal Testing, are conducted) we also assessed the AUC-ROC at 8–11 weeks gestation. At a mean of 9.4 ±1.0 gestation, the AUC-ROC for spontaneous preterm birth prediction was 0.94; p<0.0001 (Table 5).

**Table 3:** Spontaneous preterm birth risk assessment using 8 microRNAs to calculate Risk Scores in 39 patients.

Patient	miR 1267	≤ 14.3	miR 148a	≤ 28.0	miR 181a	≤ 25.8	miR 210	≤ 21.0	miR 223	≤ 12.3	miR 301a	≤ 25.1	miR 340	≤ 21.3	miR 671	≤ 28.8	#days preg	Risk Score	Delivery outcome
**1**	15.4				25.3	x	23.5		12.5		28.4		24.8		29.9		68	**1**	FULL TERM
**2**	13.7	x	30.3		27.3		22.1		10.3	x	25.4		22		28.1	x	54	**3**	FULL TERM
**3**	15.3		30.9		26.1		19.9	x	12.5		24.7	x	21.4		29.3		88	**2**	FULL TERM
**4**	16.4		35.7		27.3		23		13.7		26.4		24.3		33.8		39	**0**	FULL TERM
**5**	16.5		35.3		26.2		22.8		13.4		26.5		23.6				59	**0**	FULL TERM
**6**	15.6		33.9		24.6	x	22.1		13.7		26.8		23.5		29.6		63	**1**	FULL TERM
**7**	16.6		38.2		26.7		21.3		13.5		26.9		23.9		30.5		29	**0**	FULL TERM
**8**	14.7		30.7		25.3	x	22.2		11.9	x	24.1	x	21.8		29.2		46	**3**	FULL TERM
**9**	12	x	29.3		28.3		18.8	x	10.2	x	23.2	x	22.2		30.6		78	**4**	FULL TERM
**10**	15.8		39.2				23.2		12.7		31.2		26.7		29.8		104	**0**	FULL TERM
**11**	11.6	x					22.6		10.7	x	23.9	x	24.1		30.3		31	**3**	FULL TERM
**12**	15.1		32.5		26.1		22.5		13.7		25.3		23.3		30.4		77	**0**	FULL TERM
**13**	15.8		37.7		28.4		22.4		13.5		27.3		26.4		30.6		55	**0**	FULL TERM
**14**	14.7		35.2		27.7		25.2		12.6		29.1		25.1		32.9		68	**0**	FULL TERM
**15**	15.1		31.9		29.7		24.5		12.9		27.4		23.9		30.4		67	**0**	FULL TERM
**16**	14.1	x	34.5		24.8	x	22.1		12.4		24.6	x	22.3		28.2	x	58	**4**	FULL TERM
**17**	13.3	x	26.1	x	32.6		19.7	x	12.3	x	24.2	x	24.9		25.3	x	89	**6**	FULL TERM
**18**	13.3	x	29.8		24.7	x	21.7		11.5	x	24	x	22.1		26.9	x	47	**5**	FULL TERM
**19**	15.6		32.1		32.6		23.5		12.2	x	26.4		21.6		28.6	x	34	**2**	FULL TERM
**20**	16.1		38.7		31.2		22.8		14.2		27.8		25		32.7		41	**0**	FULL TERM
**21**	15.5		35		26.8		29.2		11.7	x	28.4		24		27.3	x	39	**2**	FULL TERM
**22**	15.8		30.9		27.7		24.6		11.8	x	26.4		24.4		31.8		33	**1**	FULL TERM
**23**	17.9		32.3		26.9		21.1		12.6		27.2		23.6		31.4		52	**0**	FULL TERM
**24**	14.8		32.5		24.7	x	22.6		12.6		25.1	x	22.7		29		69	**2**	FULL TERM
**25**	16.2		39.2		26.4		24.1		13.5		30.2		26.2		30.7		70	**0**	FULL TERM
**26**	12.8	x	28	x	23.5	x	19.7	x	9.9	x	21.9	x	20.6	x	26.5	x	38	**8**	PRETERM LATE
**27**	15.5		31.8		26.9		22.2		12.1	x	24.6	x	22.8		34.1		77	**2**	PRETERM LATE
**28**	14.6		28.9		24.3	x	18.4	x	12.1	x	23.2	x	21.8		28.2	x	84	**5**	PRETERM LATE
**29**	14.4		28.5		24.4	x	19.3	x	11.5	x	23.3	x	22.1		29.1		60	**4**	PRETERM LATE
**30**	12.9	x	25.9	x	22.8	x	18.4	x	11.2	x	22	x	20.6	x	27.7	x	37	**8**	PRETERM LATE
**31**	13	x	25	x	22.9	x	18.9	x	11.3	x	21.8	x	21	x	27.7	x	56	**8**	PRETERM LATE
**32**	13.3	x	28.5		23.1	x	21.4		10.5	x	21.8	x	19.8	x	27.1	x	21	**6**	PRETERM LATE
**33**	11	x	25.7	x	24.6	x	19.7	x	9	x	21.4	x	20.5	x	28.8	x	39	**8**	PRETERM EARLY
**34**	12.7	x	27.9	x	25.8	x	21	x	9.4	x	23.5	x	21.3	x	32.1		93	**7**	PRETERM EARLY
**35**	12.6	x	27.9	x	23.3	x	20.4	x	10.2	x	21.5	x	20.6	x	27	x	35	**8**	PRETERM EARLY
**36**	14.2	x	27.9	x	25.8	x	21.8		12.6		25.1	x	24.7		28	x	34	**5**	PRETERM EARLY
**37**	14.1	x	27.4	x	24	x	20.2	x	11.8	x	23.9	x	21.3	x	27.4	x	35	**8**	PRETERM EARLY
**38**	14.3	x	27.3	x	24.1	x	19.5	x	12.3	x	22.9	x	23		27.3	x	62	**7**	PRETERM EARLY
**39**	14.5		26.2	x	24.3	x	20.2	x	13.1		23.7	x	22.1		26.1	x	36	**5**	PRETERM EARLY

MicroRNA PCR Ct cut-off values for eight microRNAs were applied to each patient (designated in rows).

A point was given for each microRNA level where the result was less than the threshold value (each “x” designated cell).

For each patient sample, the points for each microRNA were summed together to create a Risk Score.

An AUC-ROC curve analysis was performed using these Risk Scores for SPTB prediction.

**Table 4:** AUC-ROC calculations for spontaneous preterm birth (SPTB).

	Early and Late SPTB <38 weeks)	Early SPTB (<34 weeks)	Late SPTB (34-<38 weeks)
	A	B	C
**Mean gest. age at sample collection**	7.9±3.0 weeks	8.0±2.9 weeks	8.2±2.9 weeks
**AUC-ROC**	0.95	0.98	0.92
**AUC-ROC 95% Confidence Interval**	0.83–1.00	0.86–1.00	0.77–0.99
**Preterm (14 total)**	14	7	7
**Full-term (25 total)**	25	25	25
**Sensitivity**	86	100	86
**Specificity**	92	92	84
**P value**	P<0.0001	P<0.0001	P<0.0001

**Table 5:** AUC-ROC scores for SPTB with increasing gestational age of sample collection.

Gest. age range at sample collection	4–5 weeks	6–9 weeks	9–13 weeks	Subgroup of interest: 8–11 weeks (NIPT time frame)
**#samples**	14	13	14	13
**Mean gest. age at sample collection (± SD)**	4.9±0.7 weeks	7.9±1.1 weeks	11.2±1.7 weeks	9.4±1.0 weeks
**AUC-ROC**	1	0.95	0.88	0.94
**AUROC-ROC 95% Confidence Interval**	0.77–1.00	0.68–1.00	0.60–0.99	0.67–1.00
**Preterm**	8	3	3	4
**Full-term**	6	10	11	9
**Sensitivity**	100	100	100	100
**Specificity**	100	80	64	78
**P value**	P<0.0001	P<0.0001	P = 0.0002	P<0.0001

## Discussion

We are the first to identify a biomarker panel that can successfully predict preterm birth from the early first trimester of pregnancy. Previous studies have interrogated microRNA in plasma during the first trimester of pregnancy with limited success [16, 19, 20]. Ours is the first to use microRNA in peripheral blood immune cells. Though our study size is small, results are novel and unexpected and carry many implications for the field.

Successful prediction of preterm birth during the first trimester and in particular early during the first trimester is not unforeseen. The biomarker panels identified in our previous studies similarly predicted preeclampsia and miscarriage using first trimester PBMC microRNA collected at 7–8 weeks gestation [17, 18]. Romero et al. conclude that disorders of deep placentation are a major mechanism of disease in late pregnancy which include preeclampsia and intrauterine growth restriction [4]. The authors subsequently note that, unlike preeclampsia and intrauterine growth restriction, preterm birth can involve additional mechanisms such as infection, stress, trauma and cervical disease. They suggested that preterm birth represents the endpoint for a multiplicity of causes [5]. Thus, it is surprising in our study, that quantification of microRNA isolated from peripheral blood cells collected during the first trimester should be predictive of a condition with such a broad range of contributing factors. However, our patient population was well vetted for many preterm delivery risk factors. The full term and preterm delivery groups were similar in terms of age, history, infection, autoantibody incidence and use of IVF and donor egg, excepting for BMI which was higher in the preterm birth group, (p<0.01; Table 1). All women included in the study were of a mature reproductive age (> 28 years), were in stable partner relationships and were actively seeking pregnancy. There were with no active drinkers, smokers, recreational drug users or teenage or single mothers included in the patient population. Except for the higher incidence of miscarriage and autoimmunity inherent to the clinic population, our patients represent a group where other known preterm birth co-morbidities [21, 22, 23] have been significantly reduced.

Quantification of microRNA holds promise in both detection and monitoring of disease processes. MicroRNAs interact with mRNAs encoding proteins regulating pathways fundamental to cell responses. They are released by cells into plasma permitting phlebotomy to act as a “liquid biopsy” permitting assessment of processes otherwise largely unreachable by non-invasive means. However, prior attempts to utilize microRNA in plasma as an early diagnostic in pregnancy have been disappointing [16].

Romero et al. has suggested that the Great Obstetric Syndromes share a common origin in the placental bed [4, 5]. However, until blood flow is fully established in the intervillous space at the beginning of the second trimester, the placentas of normal pregnancy and compromised pregnancy experience the same histiotrophic nutrition and low oxygen saturation. Therefore, the early placenta should not be expected to release diagnostically different quantities of microRNAs into plasma until after intervillous blood flow is fully established. Attention should, therefore, be drawn to the first trimester placental bed for the purposes of risk assessment [24, 25]. However, acquiring biomarkers from the placental bed in the first trimester is invasive.

Immune cells of the placental bed are functionally and phenotypically distinct from immune cells circulating in the peripheral blood. Immune cells within the placental bed, in particular, the decidual natural killer cell (dNK cell), support transformation of the spiral arteries [26]. Conversely, NK cells in the peripheral blood are largely cytotoxic. It is not, therefore, surprising that interrogation of peripheral blood immune cells has been regarded as a poor surrogate for the state of immune cells within the decidua [27, 28]. MicroRNAs constitute a large repertoire of biomarkers that can be identified in peripheral blood immune cells. We hypothesize that some portion of the large repertoire of microRNAs now recognized might comprise a group shared by dNK cells in the placental bed. In our previous studies, we were able to identify differentially-expressed first trimester PMBC microRNAs in women at high risk of developing preeclampsia and miscarriage [17, 18]. Several of our panel’s microRNA differentially expressed in peripheral blood cells are known to regulate processes within the decidua. Two of the eight microRNAs in our panel that predict preterm birth include miR-223 and 340 have been shown to be associated with natural killer cell inhibition [29] and maternal immune tolerance to pregnancy at the implantation site [30]. In addition, we know that decidual immune cells can be directly involved with the implantation process [31, 32, 33]. It is interesting to note that the miR-340-5p/miR-340-3p cluster associated with natural killer cells can negatively regulate cell proliferation via induction of apoptosis [34]. Enquobahrie et al. found that miR-210 and mir-223 were associated with increased risk of preterm delivery. Interestingly, miR-223 has been shown to regulate genes involved with inflammation, immune cell function, and activation [35, 36]. MicroRNA-210 has been shown to regulate the hypoxic state associated with the angiogenesis at the implantation site [37].

Unlike others who have used microRNAs released into plasma, we have shown that microRNA quantified from peripheral blood cells predicts preterm delivery risk during most of the first trimester [16, 38, 39]. Peripheral blood immune cells are recruited into the decidua where they mature within the decidual microenvironment [40, 41]. However, we note that data from our earliest sample collection, perhaps including the actual time of implantation, are predictive of clinical outcome (Table 5). We are tempted to speculate that peripheral blood immune cells may impact the placental bed, either as they mature into their decidual phenotype or directly as peripheral blood immune cells.

It is interesting to note that a few of our full term patients did experience false positive microRNA Risk scores. Two of our full term delivery patients illustrated in Table 3 (Patients #17 and 18) exceeded the designated cut-off levels for microRNAs-223 and -301. These two patients also suffered from asthma and elevated anti-phospholipid antibodies, respectively. It is interesting that both microRNAs-223 and -301a are associated with immune activation [42, 43]. To control for the possible confounding effects of an autoimmune population, it may be beneficial to design a study with a patients of low autoimmune risk in the future.

In summary, we have shown that microRNA quantified from peripheral blood cells predict preterm delivery risk during most of the first trimester. We also show that blood cell microRNA quantification exhibits excellent predictive value at 8–11 weeks, a time period when NIPT is routinely performed. Routine first trimester preterm risk assessment would for allow for earlier monitoring and intervention. Though our research is preliminary, we hope that future studies will build upon our investigations and enhance the power of maternal cell microRNA to predict pregnancy risk in the clinic.

## Supporting information

S1 File Supplementary data file containing patient data and microRNA Ct expression levels. (XLSX)
